# Morphine exposure and prematurity affect flash visual evoked potentials in preterm infants

**DOI:** 10.1016/j.cnp.2023.12.005

**Published:** 2024-01-24

**Authors:** Caterina Coviello, Silvia Lori, Giovanna Bertini, Simona Montano, Simonetta Gabbanini, Maria Bastianelli, Cesarina Cossu, Sara Cavaliere, Clara Lunardi, Carlo Dani

**Affiliations:** a Division of Neonatology, Careggi University Hospital of Florence, Florence, Italy; b Neurophysiology Unit, Neuro-Musculo-Skeletal Department, Careggi University Hospital, Florence, Italy; c Department of Neurosciences, Psychology, Drug Research and Child Health, Careggi University Hospital of Florence, Florence, Italy

**Keywords:** Visual evoked potentials, Morphine, Opioids, Preterm infants

## Abstract

**Objective:**

The present study aimed to explore first the impact of perinatal risk factors on flash-VEP waves and morphology in a group of preterm infants studied at term equivalent age (TEA). Second, to correlate VEP morphology with neurological outcome at 2 years corrected age (CA).

**Methods:**

Infants with a gestational age (GA) at birth <32 weeks, without major brain injury, were enrolled. Multivariate regression analyses were performed, and the models were run separately for each dependent variable N2, P2, N3 latencies and P2 amplitude. Logistic regression was applied to study N4 component (present/absent) and VEP morphology (regular/irregular). The predictors were GA, bronchopulmonary dysplasia (BPD), postmenstrual age at VEP registration, cumulative morphine and fentanyl dose, and painful procedures. Lastly, linear regression models were performed to assess the relation between the Bayley-III cognitive and motor scores at 2 years CA and VEP morphology, in relation to GA, BPD, painful procedures and cumulative morphine dose.

**Results:**

Eighty infants were enrolled. Morphine was the predictor of N2 (R^2^ = 0.09, *p* = 0.006), P2 (R^2^ = 0.11, *p* = 0.002), and N3 (R^2^ = 0.13, *p* = 0.003) latencies. Younger GA was associated with lower amplitude (R^2^ = 0.05, *p* = 0.029). None of the independent variables predicted the presence of N4 component, nor VEP morphology in the logistic analysis. VEP morphology was not associated with cognitive and motor scores at 2 years.

**Conclusions:**

Morphine treatment and prematurity were risk factors for altered VEPs parameters at TEA. In our cohort VEP morphology did not predict neurological outcome.

**Significance:**

Morphine administration should be evaluated according to potential risks and benefits, and dosage individually accustomed, according to pain and comfort scores, considering the possible risk for neurodevelopmental impairment.

## Highlights

• Visual evoked potentials (VEP) are an accurate method to evaluate the maturity of the central nervous system.
• Morphine treatment and prematurity were risk factors for altered VEPs parameters at term equivalent age.
• Morphine administration should be carefully pondered according to pain score in preterm infants.

## 1 Introduction

Recent advances in perinatal and neonatal care have improved the survival of preterm infants, although children born preterm are still at risk for neurodevelopmental disabilities, including motor, cognitive, hearing and visual deficits (Leung et al., 2018, Marlow et al., 2005). White matter injury (WMI), germinal matrix hemorrhage – intraventricular hemorrhage (GMH-IVH) and cerebellar hemorrhage (CH) are the most diffuse type of brain injury in survivors of premature birth (Volpe, 1989, Volpe and Zipurksy, 2001). However, even in the absence of an evident brain damage, preterm infants are at risk for impaired neurodevelopment (Montagna and Nosarti, 2016). Gestational age (GA) at birth, size for GA, postnatal growth, nutrition and neonatal morbidities such as sepsis, necrotising enterocolitis (NEC) and bronchopulmonary dysplasia (BPD) influence neurodevelopmental outcomes (Adams-Chapman et al., 2018, Ehrenkranz et al., 1999, Franz et al., 2015, Goudoever et al., 2018, Rees et al., 2007, Schlapbach et al., 2011). Moreover, on one hand, extensive data have demonstrated the adverse effect of pain and stress on short- and long-term outcome, leading to the control of pain strongly recommended in the NICU (American Academy of Pediatrics, Committee on Fetus and Newborn and Section on Surgery, Section on Anesthesiology and Pain Medicine, Canadian Paediatric Society, 2006, Boggini et al., 2021). On the other hand, concerns on opioid analgesic drugs have been widely expressed about their impact on brain development (Bellù et al., 2010, Luzzati et al., 2023, Schuurmans et al., 2015).

A precise evaluation and early investigation of brain injury is mandatory in preventing neurological impairment, and in this neuroimaging techniques play an essential role in preterm infants. Alongside the neuroimaging examinations, the use of visual evoked potentials (VEP) are an accurate and non-invasive method to evaluate the maturity and integrity of the central nervous system (Brecelj, 2003). VEPs are electrophysiological responses elicited by visual stimuli extrapolated from electroencephalographic activity recorded from the scalp overlying the occipital cortex (Odom et al., 2016). The appearance of a VEP after a flash or pattern reversal stimulus indicates that visual information has efficaciously reached and activated the cortex. This neurophysiological technique has been widely applied in preterm and term infants and several studies have highlights its usefulness for early identification of newborns at high risk of neurodevelopmental impairment (Atkinson et al., 2002, Kato et al., 2005, Kato and Watanabe, 2006, Tsuneishi et al., 1995). Indeed, infants who suffered severe brain injury, exhibit immature or abnormal or poor flash VEP waveforms (Cainelli et al., 2018, Eken et al., 1994, Kato et al., 2005, De Vries et al., 1986). Flash VEP can be recorded in infants as preterm as 24 weeks of gestation and related waveforms change with the progression of maturation of the visual pathways (Pike et al., 1999). Wave P2 is present in all healthy infants born from 37 weeks of gestational age (GA) onwards, whose latency shows a low variability making it a reliable biomarker of impaired neonatal brain maturation (Benavente et al., 2005).

In addition, VEP has also been explored for its prognostic value on neurodevelopmental outcomes in infants, however past researches reported inconclusive findings (Beverley et al., 1990, Ekert et al., 1997, Kato and Watanabe, 2006, Pike and Marlow, 2000, Shepherd et al., 1999).

The present study has two aims, first to explore the impact of perinatal risk factors on VEP waves and morphology in a group of preterm infants, without major neurologic impairments, studied at term equivalent age (TEA). Second, to correlate flash-VEP morphology with neurological outcome at 2 years corrected age (CA).

## 2 Methods

### 2.1 Study population

A prospective observational cohort study was conducted between September 2018 and May 2021 at Careggi University Hospital of Florence. Infants with GA <32 weeks, born and admitted to the third level NICU of our hospital surviving to term equivalent age (TEA) were eligible for recruitment. Written parental informed consent and permission from the local ethics committee medical were obtained for this study (112/2018).

Exclusion criteria were the presence of major congenital malformations or syndromes, transfer to other hospitals, or death before 36 weeks of postmenstrual age (PMA). Infants with severe brain injury, defined as the occurrence of intraventricular hemorrhage (IVH) ≥ 3 grade (Papile et al., 1978) and cystic periventricular leukomalacia (PVL) (de Vries et al., 1992) were also excluded, since previous works reported immature or abnormal flash VEP waveforms in the these patients (Eken et al., 1994, Kato et al., 2005, De Vries et al., 1986). For the same reason, patients with retinopathy of prematurity (ROP) ≥ 3 grade were not considered (Mintz-Hittner et al., 1994).

### 2.2 Perinatal data

Medical chart review was carried out and for each infant the following perinatal data were obtained: GA, birth weight, gender, type of delivery, need and duration of mechanical ventilation, postnatal steroids, occurrence of BPD (Davidson and Berkelhamer, 2017), patent ductus arteriosus (PDA), culture-proven sepsis, NEC (Bell et al., 1987), grade of IVH (Papile et al., 1978), PVL (de Vries et al., 1992) and ROP (Quinn, 2005). Birth weight and head circumference z-scores were computed according to the INES charts (Bertino et al., 2010).

2.3 Pain evaluation and management

The exposure to pain was evaluated by recording the number of the following invasive procedures from birth to neurophysiological examination: heel pricks, peripheral or central venous catheter insertion, endotracheal intubation, intra-muscular injection, chest tube insertion, and urinary catheter insertion, lumbar puncture, and eye examination. For subsequent analyses, infants were grouped into low (lowest 50 %) or high (highest 50 %) painful procedures exposure, according to the median value. The cumulative dose of fentanyl (intravenous dose) and morphine (intravenous dose plus converted oral dose) were calculated as the average daily dose adjusted for patient’s weight during the NICU stay. In our NICU, opioids, such as fentanyl and morphine, were used in mechanically ventilated infants, according to pain scores and clinical indications, as suggested by the guideline of the Italian Society of Neonatology. Fentanyl was administrated as first choice and, in case of prolonged ventilator dependence and to avoid fentanyl tolerance or tachyphylaxis, morphine was introduced. The shift was usually performed between the tenth and the fourteenth day of fentanyl therapy. Infants could receive fentanyl as an intravenous bolus (1–3 μg/Kg) or continuous intravenous infusion (0.5–3 μg/Kg/h) as necessary. Morphine was administered intravenously starting with a loading dose (0.05–0.1 mg/kg/dose) followed by a continuous infusion (0.01–0.05 mg/kg/h), and when the infant was at full enteral feeding was orally dispensed (Lago et al., 2006).

### 2.4 Visual evoked potentials recordings

VEP recordings were performed using Nemus-EB Neuro polygraph and GalNT / EP EXAM software, which provided also the recording of VEEG. VEPs were recorded during active sleep state, while the newborns were lying in their cot in a supine position in a dark room.

All infants were clinically stable, and none received drugs influencing brain activity, such as morphine, benzodiazepines, and barbiturates during the exam.

Electrode landmarks were identified at the level of the occipital scape according to the International System 10–20 (Gilmore, 1994, Society, 2008). Disposable surface electrodes in O1-O2-Oz were placed with reference Fz and impedances <10 kΩ; the Flash-Lamp stimulator (stimulus surface 12x4 cm) was positioned at 30 cm from the neonate's eyes and flashes of light at low frequency of 1 Hz were sent to obtain a transient response. The recording parameters were analysis time 1 s, band pass filter 1–200 Hz, sensitivity 50 µV. To avoid habituation related to high stimulation rate and high number of stimuli, and to ensure reproducibility of the traces, the average consisted of two series of 30 responses.

**Fig. 1:**
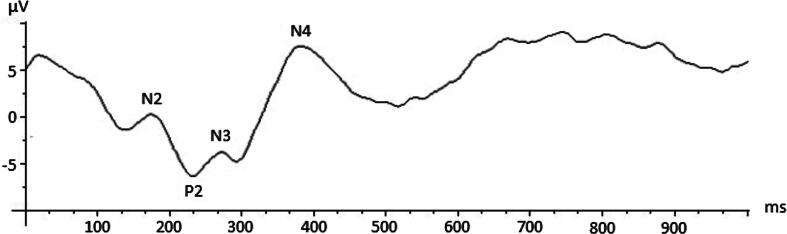
Flash VEP waveforms showing VEP components in a full- term infant.

**Fig. 2:**
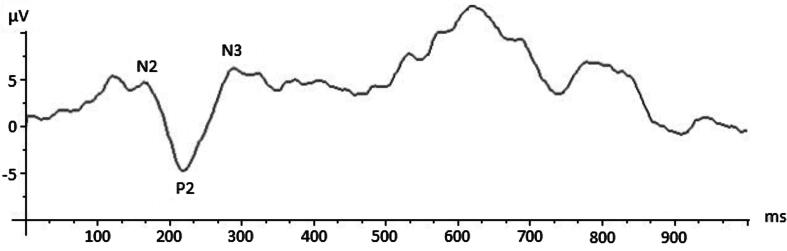
An example of Flash VEP waveforms with atypical morphology.

The flash VEP waveforms obtained in our population consisted of four separate phases, a first negative deflection named N2, followed by a prominent positive wave called P2, a later negative deflection named N3, and when present a further negative deflection named N4 (Fig. 1). Latencies of N2, P2, N3 were measured, as P2 amplitude. N4 wave was categorized as present or absent. Each flash VEP was also categorized by waveform morphology. Descriptive categories were: regular, immature, atypical, not detectable (Benavente et al., 2005, McGlone et al., 2008), (Fig. 2). For the analysis the morphology was labeled as regular and irregular. Good quality VEP was defined as reproducible response for at least three series of acquisition, without excessive artifact.

### 2.5 Neurodevelopment assessment

Neurodevelopmental outcome was evaluated at 2 years corrected age (CA) using the Bayley Scales of Infant and Toddler Development, 3rd edition (BSITD-III). Cognitive and motor composite scores were calculated (mean in a normative population 100, standard deviation 15).

### 2.6 Statistical analysis

The infants’ clinical characteristics were described as mean and standard deviation, median and interquartile range (IQR) or rate and percentage. The multivariate regression analyses with backward elimination were performed to assess the association between VEP (as dependent variables) and clinical risk factors. The models were run separately for each dependent variable N2 latency, N3 latency, P2 latency and amplitude. Our models contained the following predictors: GA, BPD, PMA at VEP registration, cumulative fentanyl and morphine dose, painful procedures (high/low exposure). Logistic regression was executed for N4 component (present/absent) and VEP morphology (regular/irregular), adjusting for the previous mentioned variables. Pearson correlation did not show an association between P2 latency and GA, PMA, BPD and painful procedures (all p > 0.1) (Supplementary Table 1). However, GA, size at birth and PMA were entered because a number of investigators have reported their effect on VEP latencies and morphology (Hrbek et al., 1982, Pike et al., 1999, Schwindt et al., 2018, Stanley et al., 1991, Thordstein et al., 2004, Tsuneishi et al., 1995, Tsuneishi and Casaer, 2000, Watanabe et al., 1972). BPD was included because of its adverse impact on neurodevelopmental outcome (Franz et al., 2015, Goudoever et al., 2018, Schlapbach et al., 2011). Morphine and fentanyl were studied because Pearson correlation did show a positive association with P2 latency (R = 0.34, p = 0.002; R = 0.25, p = 0.021, respectively). Exposure to pain was then entered for two reason: first, because in the absence of stress or pain, opioids had a detrimental effect on brain maturation, second because its negative impact on neurodevelopment (Supplementary Table 1) (Duerden et al., 2020, Traudt et al., 2012).

In addition, infants’ data were grouped according to whether they had received morphine or not. The *t* test and chi square test were used to compare continuous normally distributed data and categorical data.

Lastly, linear regression models with backward elimination were performed to assess the relation between the Bayley-III cognitive and motor scores at 2 years CA and VEP morphology, in relation to GA, BPD, painful procedures (high/low exposure), cumulative morphine dose.

Results are presented as coefficients of independent variables with 95 % confidence intervals (CI). Condition indexes and the variance inflation factor of the regression model were computed to detect multi-collinearity. Condition Index <30 and variance inflation factor <5 values indicated that regression models did not have significant multicollinearity. Data analysis was performed using IBM SPSS Statistics version 20.

## 3 Results

### 3.1 Subjects

Table 1 shows the baseline characteristics of the preterm infant cohort. F-VEPs of 80 preterm infants were analyzed. VEPs were recorded at 39.3 ± 1.2 weeks of PMA and at a mean weight of 2238 ± 363 gr. The VEPs characteristics are presented in Table 2.

**Table 1:** Clinical characteristics of studied infants. Mean ± SD, median and (IQR), or rate and (%).

	N = 80
Gestational age (wks)	27.9 ± 1.9
Male	46 (57)
Birth weight (gr)	1009 ± 307
Birth weight, Z score	0.07 ± 0.90
Head circumference at birth (cm)	25.2 ± 2.5
Head circumference, z score at birth	−0.16 ± 1.13
Caesarean section	58 (72)
Apgar score at 5 min	8 (8–8)
Mechanical ventilation	33 (50)
Mechanical ventilation duration (d)	8 (3–20)
NICU stay (d)	85 ± 25
PMA at VEP registration (wks)	39.3 ± 1.2
Weight at VEP registration (g)	2238 ± 363
Weight z-score at VEP registration	−2.20 ± 1.24
Head circumference at VEP registration (cm)	31.8 ± 1.6
Head circumference z-score at VEP registration	−1.7 ± 1.6
PDA requiring treatment	54 (67)
NEC	2 (3)
BPD	29 (36)
Sepsis	42 (52)
IVH	
*grade I*	7 (11)
*grade II*	1 (2)
ROP	
*grade 1*	7 (11)
*grade 2*	0 (0)
Postnatal steroids	21 (26)
N° painful procedures	90 (66–131)
Morphine exposure (n)	13 (16)
Dose (mg/kg)	15.9 (5.6–23)
Duration (d)	35 (20–53)
Fentanyl exposure (n)	54 (67)
Dose (mcg/kg)	3.2 (1.5–243)
Duration (d)days	2.5 (1–10)
Cognitive scores at 2 years CA	92 ± 8
Motor scores at 2 years CA	87 ± 5

PMA, post menstrual age; VEP, visual evoked potentials; PDA, patent ductus arteriosus; NEC, necrotizing enterocolitis; BPD, bronchopulmonary dysplasia; IVH, intraventricular hemorrhage; ROP, retinopathy of prematurity; CA, corrected age.

**Table 2:** Flash VEP characteristics of preterm infants. Mean ± SD or rate and (%).

	**N = 80**
**N2 latency (msec)**	170.5 ± 25.7
**P2 latency (msec)**	210.1 ± 24.8
**N3 latency (msec)**	260.8 ± 40.1
**N4 component presence**	11/69 (14 %)
**VEP Morphology**	
**regular**	67 (84 %)
**irregular**	
***immature***	8 (10 %)
***atypical***	5 (6 %)
***undetectable***	0 (0)
**P2 amplitude (μV)**	7.8 ± 6.5

In our cohort fifty-four infants received fentanyl (67 %) at a median dose of 3.2 (IQR 1.2–243; range 0,5–1890) mcg/kg and for a median of 2.5 (1–10) days during their NICU stay. Thirteen infants were administered morphine (16 %) at a median dose of 15.9 (IQR 5.6–23; range 0.1–83.2) mg/kg and for a median of 35 (20–53) days.

Neonates who were exposed to morphine were ventilated for longer period, had higher incidence of IVH, BPD, ROP, compared to their pair who were not exposed (Supplementary Table 2).

### 3.2 Assessment of perinatal risk factors affecting flash-VEP among preterm infants

**Fig. 3:**
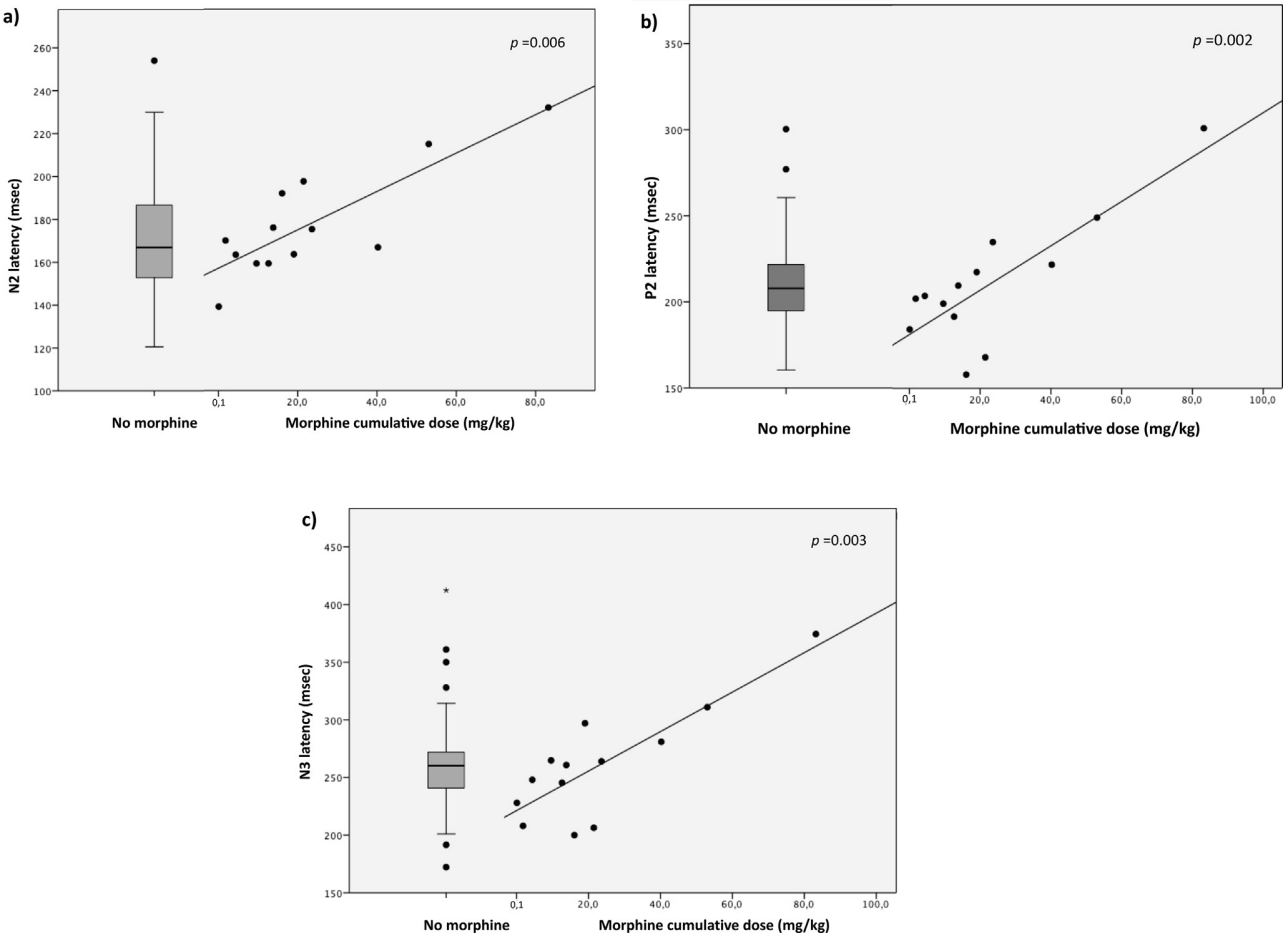
Correlation between cumulative morphine and N2 (**a**), P2 (**b**), and N3 (**c**) latencies. Multivariable regression analysis.

The multivariate analyses showed that cumulative morphine was the predictor of N2 (R^2^ = 0.09, *p* = 0.006), P2 (R^2^ = 0.11, *p* = 0.002), and N3 (R^2^ = 0.13, *p* = 0.003) latencies in the model built with GA, birth weight (z-score), PMA, BPD, cumulative fentanyl dose and pain exposure as covariates (Table 3, Fig. 3a, b, c). The results of the regressions indicated that the model for N2, P2 and N3 latencies explained 9 %, 11 %, and 13 % of the variance, respectively, and 1 extra mg/kg of morphine increased N2, P2 and N3 latencies of 0.63, 0.94, 1.10 msec, respectively.

**Table 3:** Multivariate linear regression analyses (backward elimination) between flash VEP and clinical risk factors.

	N2 Latency (msec)			P2 Latency (msec)			N3 Latency (msec)			P2 amplitude (μV)		
	**Adjusted R ^2^** = 0.09			**Adjusted R ^2^** = 0.11			**Adjusted R ^2^** = 0.13			**Adjusted R ^2^** = 0.05		
	**B**	**95 % CI**	***p***	**B**	**95 % CI**	***p***	**B**	**95 %CI**	***p***	**B**	**95 %CI**	***p***
GA			−			−	4.4	0.01–8.9	0.059	0.78	−0.08–1.48	**0.029**
Birth weight (z-score)			−			−			−			−
PMA at registration			−			−			−			−
BPD			−			−			−			−
Painful procedures			−			−			−			−
Fentanyl cumulative dose (mcg/kg)			−			−			−			−
Morphine cumulative dose (mg/kg)	0.63	0.19–1.07	**0.006**	0.94	0.38–1.50	**0.002**	1.10	0.39–1.8	**0.003**			−

GA, gestational age; PMA, post menstrual age; BPD, bronchopulmonary dysplasia.

**Fig. 4:**
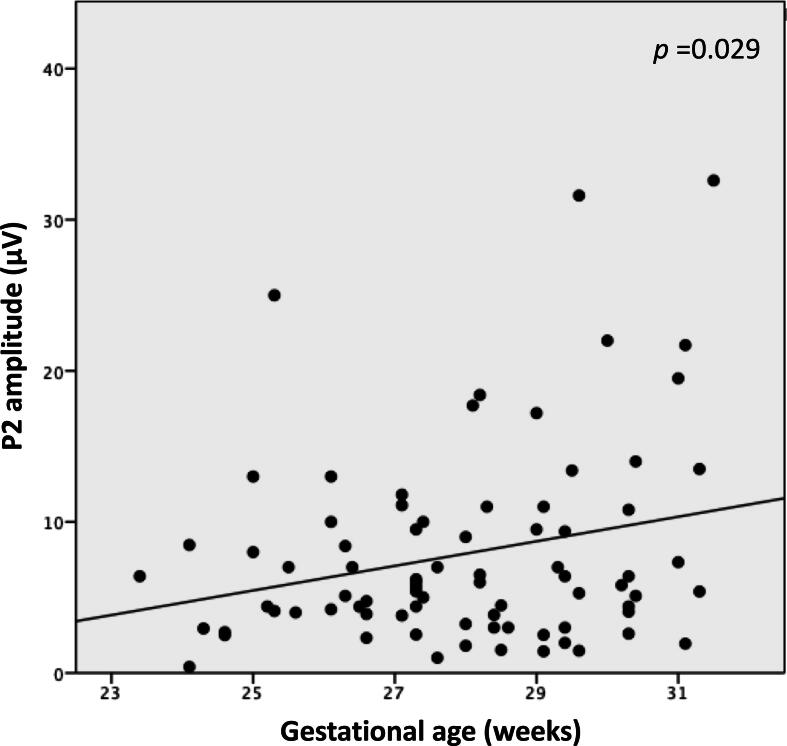
Correlation between gestational age and P2 amplitude. Multivariable regression analysis.

Younger GA was associated with lower amplitude (R^2^ = 0.05, *p* = 0.029) (Table 3, Fig. 4); the model explained 5 %, of the amplitude variance.

None of the independent variables predicted the presence of N4 component, nor the irregular morphology in the logistic analysis (Table 4).

**Table 4:** Logistic regression analysis (backward elimination) evaluating the possible correlation between presence of N4 component and VEP morphology, and clinical risk factors.

	N4			VEP morphology		
	**OR**	**95 % CI**	**p**	**OR**	**95 % CI**	***p***
GA			−			−
Birth weight (z-score)			−			−
PMA at registration			−			−
BPD			−			−
Painful procedures (high/low exposure)	2.5	0.6–9.2	0.159	3.6	0.8–15.2	0.071
Fentanyl cumulative dose (mcg/kg)			−			−
Morphine cumulative dose (mg/kg)			−			−

GA, gestational age; PMA, post menstrual age; BPD, bronchopulmonary dysplasia.

### 3.3 Assessment of VEP morphology affecting cognitive and motor outcomes

The mean scores for cognitive was 92 ± 8, and for motor was 87 ± 5 outcomes. Exploring the relationship between VEP morphology and Bayley III examination, morphology at TEA did not predict any outcome (Table 5).

**Table 5:** Multivariate linear regression analyses (backward elimination) between flash VEP morphology and neurological outcome at 2 years CA.

	Cognitive outcome			Motor outcome		
	**Adjusted R ^2^** = 0.11			**Adjusted R ^2^** = 0		
	**B**	**95 % CI**	***p***	**B**	**95 % CI**	***p***
GA	−1.5	−3.4––0.2	0.093			−
BPD			−			−
Painful procedures	−5.4	−11.7–0.8	0.088			−
Morphine cumulative dose (mg/kg)			−			−
VEP Morphology			−			−

GA, gestational age; BPD, bronchopulmonary dysplasia.

## 4 Discussions

The first aim of our study was to investigate the effect of perinatal factors on F-VEPs. We demonstrated that a higher cumulative morphine dose predicted longer N2, N3, and P2 latency at TEA, and that earlier GA were associated with lower P2 amplitude. These correlations were found to be independent from other clinical risk factors since they persisted after adjusting for variables reflecting neonatal morbidities.

Opioid analgesics drugs are given to critically ill infants in an effort to diminish pain and stress induced by procedures and mechanical ventilation. Preclinical studies have demonstrated that untreated repetitive pain and stress can activate N-methyl-D-aspartate (NMDA) receptors, increasing hypoxia-induced glutamate liberation and excitotoxicity (Johnston et al., 2002). On the other hand, the impact of opioids on neurodevelopment is not clear. Many authors indicated a detrimental effect of morphine on early brain activity, brain volumes and cerebellum volumes at TEA (Al-Mouqdad et al., 2022, Van Den Bosch and Marroun, 2015, Tataranno et al., 2020, Zwicker et al., 2016). However, results on neurodevelopment were inconclusive in demonstrating advantage or disadvantage of morphine administration (Ferguson et al., 2012, De Graaf et al., 2013, De Graaf et al., 2011, Roze, 2015).

Opioids can also impact the visual system. Studies in animal models have reported that opiate receptors are present in the visual pathways (Lewis et al., 1983, Walker et al., 1988, Wise and Herkenham, 1982). Chronic morphine exposure caused the degradation of the receptive field of lateral geniculate nucleus cells (He et al., 2005) and the decrease in total dendritic length and spine density in the visual cortex (Li et al., 2007). Furthermore, prenatal exposure to morphine has been shown to reduce the total length of the basal dendrites of the layer II/III in the lateral secondary visual area (Mei et al., 2009).

Studies in humans have shown that infants prenatally exposed to opioids exhibited smaller network volumes particularly in the primary visual network compared to their unexposed pairs (Merhar et al., 2021). Furthermore, a long term follow-up research revealed that children randomized to early morphine infusion exhibited lower scores at the visual analysis subtest of IQ at 5 years of age, compared to unexposed infants (De Graaf et al., 2011).

Opioid receptors are expressed in the human brain at cortex level, lateral geniculate nucleus, and midbrain (Robinson and Kolb, 1999). The opioid system is crucial for neuronal maturation and modulates GABAergic synaptic transmission. Excitatory and inhibitory GABA synaptic transmission, in turn, is fundamental for the development and maintenance of visual cortical function (Cruz et al., 2004, Laviolette et al., 2004).

Latencies of F-VEPs have been correlated to white matter maturation (Tsuneishi and Casaer, 1997) and, specifically, prolonged latencies have been associated with poorer myelination (You et al., 2011). Thus, the increased latency found in our patients might reflect a delayed myelination of the primary visual pathways induced by morphine administration.

Previous research have shown that the endogenous opioid system may also play an important role on myelination process (Sanchez et al., 2008). Neural stem cells and oligodendroglial lineage cells express opioid receptors and their modulation in culture has been reported to have mitogenic and differentiating properties (Eschenroeder et al., 2012, Knapp et al., 1998, Persson et al., 2006). These findings suggest that any opioid treatments or abuse might interfere with endogenous opioid system influencing brain myelination. A recent neuroimaging study further support this theory, demonstrating that morphine exposure in the first week of life was associated with a high incidence of white matter injury (WMI) with a dose dependent association (Al-Mouqdad et al., 2022). The NEOPAIN trial, as well, showed that open-label morphine administration was correlated with PVL, in addition to IVH, and/or neonatal death (Anand et al., 2004).

Interestingly, our findings indicate that morphine may affect the visual function, but not fentanyl. Notwithstanding, only 16 % of patients in our cohort received morphine, the median cumulative morphine dose and the days of exposure were higher comparing to other researches about morphine and brain development (Doyle and Inder, 2015, De Graaf et al., 2011, Luzzati et al., 2023, Zwicker et al., 2016). In our NICU, pain was managed in agreement with the guideline of the Italian Society of Neonatology (Lago et al., 2006) and morphine was a second choice drug. Infants who received morphine, had received fentanyl before, were sicker and intubated for longer period compared to their pairs not exposed, needed high dose of morphine to control pain and stress and avoid abstinence syndrome. Thus, our findings might be partially induced by the high dose of morphine received by our patients.

Interestingly our findings indicated that P2 amplitude was predicted by GA. Amplitudes of the response components have been related to synaptogenesis in the visual cortex and axonal growth (Chayasirisobhon et al., 2012, Dubois et al., 2008, Scherg and Picton, 1991, Tsuneishi and Casaer, 1997). According to ongoing maturation, P2 amplitude showed a progressive increase in preterm infants. Previous studies have shown that infants born preterm as compared to full term, exhibited lower amplitude in the first months after birth (Feng et al., 2013, Kharal et al., 2020), although authors failed to demonstrate a direct correlation with GA. Our model explained only 5 % of the variance of the amplitude and therefore, other factors not analyzed in our cohort might influence this parameter.

We could not find an association between F-VEP morphology and perinatal risk factors. As mentioned earlier, abnormal VEP morphology has been frequently described in preterm infants with IVH and PVL (Eken et al., 1996, Pike et al., 1990, Placzek et al., 1985, Pryds et al., 1989, De Vries et al., 1986). Previous studies reported a variety of anomalous flash VEPs in infants born preterm but all of those studies included children with brain injury, in contrast with our study, where none had severe brain damage and only 13 % showed low grade IVH. In addition, abnormal visual electrophysiology was a frequent finding in infants prenatally exposed to opioids. Earlier studies have demonstrated that VEPs of infants prenatally exposed to methadone were more likely to have immature waveform and to be smaller in overall amplitude at birth in comparison to unexposed infants (McGlone et al., 2008, McGlone et al., 2013) The possible explanation for these conflicting findings likely reflect the fact that participants in our study received adequate dose of opioids and for the necessary time in order to control pain and discomfort, while in McGlone’s study, infants were born from drug abusing mother, thus the dose and the time of exposure were not under control.

Statistical analysis did not find significant association between N4 component and perinatal risk factors. N4 component is a primitive wave that can be evoked as early as 24 weeks GA (Pike et al., 1999, Shepherd et al., 1999). N4 latency decreased with increasing GA at birth (Stanley et al., 1991) This wave is preceded or replaced in the healthy term newborn by the positive wave P2 as result of the neuronal maturation of the neonatal visual cortex (Taylor et al., 1987). Thus, its persistence indicates a delay maturation of visual pathways. Our cohort is composed by relatively “healthy” preterm newborn and overall, N4 component was recorded in only eleven newborns (14 %), thus this might be a possible explanation of why it was not possible to find an association between N4 component and perinatal risk factors.

Additionally, we failed to find a correlation between VEP and neurodevelopmental outcome at 2 years CA. Our findings aligned with two other researches (Beverley et al., 1990, Ekert et al., 1997). On the contrary, Shepherd and Pike indicated VEP as a good prognostic indicator of neurodevelopmental delay, cerebral palsy or death, however, infants with severe brain injury [c-PVL, severe IVH (grade III or IV) were included in these studies (Pike and Marlow, 2000, Shepherd et al., 1999). The lack of this correlation might reflect the characteristic of our cohort, where most of the infants (84 %) exhibited regular VEP morphology, none suffered of severe brain injury and at follow up examination cognitive scores were in a regular range. As far as it concern the motors scores, the values were in a lower range. Although only 13 % of the infants exhibited low grade IVH a more detailed evaluation of the white matter might help explaining this finding.

Limitations of this study include the small sample size, which might have limited the possibility of detecting a significant role of other comorbidities and variables. Second, in our cohort only a minority of the infants received morphine and, therefore, we could not draw conclusion on its negative effect. Third, the study was conducted at a single center, hence reflects the treatment decisions about opioids made by the neonatologist on their clinical experience and on the protocol for pain management. Fourth, we speculated that the increased in F-VEPs latencies might indicated a delayed myelination of the primary visual pathways in morphine exposed infants, but we were not able to evaluate the myelination with neuroimaging studies. Fifth, the lack of a term control group, useful to extrapolate normative data.

## 5 Conclusions

Overall, this study showed that morphine treatment and prematurity were risk factors for altered VEPs parameters at TEA. Morphine administration should be carefully pondered and dosage individually accustomed, according to pain and comfort scores, considering the possible risk for neurodevelopmental impairment. In our cohort composed of preterm infants without major brain injury, VEP morphology did not predict neurological outcome. Additional prospective researches on larger population of infants are needed to better define the role of morphine and the possible predictive value of flash VEP on brain development.

## CRediT authorship contribution statement

**Caterina Coviello:** Conceptualization, Methodology, Data curation, Writing – original draft. **Silvia Lori:** Conceptualization, Methodology, Writing – original draft. **Giovanna Bertini:** Conceptualization, Methodology, Writing – original draft. **Simona Montano:** Data curation. **Simonetta Gabbanini:** Data curation. **Maria Bastianelli:** Data curation. **Cesarina Cossu:** Data curation. **Sara Cavaliere:** Data curation. **Clara Lunardi:** Data curation. **Carlo Dani:** Conceptualization, Methodology, Writing – original draft.

## Declaration of competing interest

The authors declare that they have no known competing financial interests or personal relationships that could have appeared to influence the work reported in this paper.
