# Supplementary material for: Morphine exposure and prematurity affect flash visual evoked potentials in preterm infants
Source: Clin Neurophysiol Pract. 2024 Jan 24;9:85–93. doi: 10.1016/j.cnp.2023.12.005 (PMC10869246; doi:10.1016/j.cnp.2023.12.005)
Supplement: Supplementary data 1 [file mmc1.docx]

**Supplementary table 1.** Pearson correlation between P2 latency and clinical risk factors.

|  | P2 latency | |
| --- | --- | --- |
|  | **R** | ***p*** |
| Gestational age | -0.06 | 0.550 |
| Birth weight (z-score) | -0.01 | 0.923 |
| PMA at registration | -0.07 | 0.510 |
| Bronchopulmonary dysplasia | 0.02 | 0.827 |
| Painful procedures (high/low exposure) | 0.04 | 0.705 |
| Fentanyl cumulative dose (mcg/kg) | 0.25 | **0.021** |
| Morphine cumulative dose (mg/kg) | 0.34 | **0.002** |

PMA, post menstrual age.
