# Supplementary material for: Morphine exposure and prematurity affect flash visual evoked potentials in preterm infants
Source: Clin Neurophysiol Pract. 2024 Jan 24;9:85–93. doi: 10.1016/j.cnp.2023.12.005 (PMC10869246; doi:10.1016/j.cnp.2023.12.005)
Supplement: Supplementary data 2 [file mmc2.docx]

**Supplementary table 2.** Clinical characteristics of the studied infants grouped according to whether they had received morphine or not. Mean ± standard deviation (SD), Median (IQR) or rate (%).

|  | **No Morphine**  **(N 67, 84 %)** | **Morphine**  **(N 13, 16 %)** | ***p value*** |
| --- | --- | --- | --- |
| GA, (weaks), mean ± DS | 28.2 ± 1.7 | 26.4 ± 2.5 | 0.052 |
| Male, n (%) | 37 (53) | 9 (69) | 0.473 |
| Birth weight (gr), mean ± DS | 1039 ± 290 | 843 ± 366 | 0.472 |
| Birth weight, Z score, mean ± DS | -0.05 ± 0.90 | -0.18 ± 0.80 | 0.666 |
| Head circumference at birth (cm), mean ± DS | 25 ± 2 | 23 ± 2 | 0.830 |
| Head circumference, z score at birth, mean ± DS | -0.13 ± 1.1 | -0.29 ± 0.7 | 0.137 |
| Caesarean section, N (%) | 52 (77) | 6 (46) | 0.066 |
| Apgar score 5 min, median (IR) | 8 (8-8) | 6.5 (7-8) | 0.462 |
| Mechanical ventilation, N (%) | 20 (32) | 13 (100) | **<0.001** |
| Mechanical ventilation days, median (IR) | 1 (0-3) | 28 (15-46) | **<0.001** |
| NICU stay (days), mean ± SD | 80 ± 25 | 113 ± 34 | 0.956 |
| PMA at VEP registration (weeks), mean ± SD | 39.3 | 39.9 | 0.492 |
| Weight at VEP registration (gr), mean ± SD | 2255 ± 367 | 2154 ± 353 | 0.494 |
| Weight z-score at VEP registration, mean ± SD | -2.07 ± 1.23 | -3.09 ±1.18 | 0.893 |
| Head circumference at VEP registration (cm), mean ± SD | 31.8 ± 1.5 | 30.8 ± 1.6 | 0.867 |
| Head circumference z-score at VEP registration, mean ± SD | -1.6 ± 1.4 | -3.4 ± 1.7 | 0.350 |
| PDA, N (%) requiring treatment | 41(61) | 13(100) | 0.056 |
| NEC, N (%) | 1 (2) | 1 (8) | 0.696 |
| BPD N (%) | 20 (30) | 9 (69) | **0.022** |
| Culture proven sepsis, N (%) | 34 (1) | 8 (61) | 0.145 |
| IVH, N (%)  *grade I*  *grade II* | 5 (7)  0 (0) | 2 (15)  1 (8) | **0.014** |
| ROP, n (%)  *grade 1*  *grade 2* | 2 (3)  0 (0) | 5 (7)  0 (0) | **<0.001** |
| Postnatal steroids, N (%) | 10 (17) | 11 (85) | **<0.001** |

GA, gestational age; PMA, post menstrual age; VEP, visual evoked potentials; PDA, patent ductus arteriosus; NEC, Necrotizing enterocolitis; BPD, bronchopulmonary dysplasia; IVH: intraventricular hemorrhage; ROP, retinopathy of prematurity.
